# Collectanea

**Published:** 1831-03

**Authors:** 


### COLLECTANEA.

**Floriferis ut apes in saltibus omnia libant,**
**Omnia nos, itidem, depascimur aurea dicta.**

### PHYSIOLOGY.

*On the Mechanism of the Act of Vomiting.* By Marshall Hall, M.D. F.R.S.E. &c. &c.

I was greatly interested by the following extract from the valuable Report of Cases in the Meath Hospital, just published by Drs. Graves and Stokes, in the fifth volume of the "Dublin Hospital Reports and Communications."

"A man, about forty years of age, died of tubercular phthisis.

"The œsophagus, after passing through the usual opening in the diaphragm, was found to re-enter the thorax by another very large opening in the tendinous portion towards the left side. The stomach occupied the inferior portion of the left thoracic cavity, its cardiac and pyloric extremities both lying in the opening.

"The man vomited frequently while under observation in the hospital. Now, as the stomach was placed entirely out of the reach of being compressed by the contractions of the diaphragm, and as this contraction completely defended it from the influence of the abdominal muscles, it is clear that, in this case, vomiting must have occurred independently of compression, either of the diaphragm or of the abdominal muscles. This fact, worth a thousand experiments, completely decides the question, that vomiting may be produced by the action of the stomach itself, unassisted by any external compressing force, notwithstanding what Le Gallois and late physiologists have said to the contrary."

The authors of the Report do not appear to have seen the paper* which I published in the number of this Journal for April to July, 1828; the object of which was, first, to expose the fallacy, both of that view of the nature of the act of vomiting which refers it to a contraction of the stomach itself, and of that other view lately advocated by M. Magendie, which refers this act to the simultaneous contraction of the diaphragm and abdominal muscles; and, secondly, to propose a new view of this disputed question. As this last view has never been controverted; as it has, on the other hand, been generally admitted; and as it alone explains the various difficulties which beset each or both of the other two, it may not be amiss to reproduce its broad outlines here, in connexion with the interesting case of Dr. Graves and Dr. Stokes. They are these:

1. During the act of vomiting, the larynx is closed;
2. The diaphragm, and its various apertures, are relaxed; and,
3. All the muscles of expiration are called into action; but,
4. Actual expiration being prevented by the closure of the larynx, the force of the effort is expended upon the stomach, the cardia being open from the relaxed condition of the diaphragm; and vomiting is effected.

It is plain, from this view of the subject, that the thorax and abdomen become one cavity, as it were, the diaphragm lying loose and inert between them. It is also obvious, that it is quite indifferent on which side of the diaphragm the stomach may be placed, whether above, as in the case of hernia, or below, in its natural situation.

The view of the act of vomiting which I have taken, appears to me to be the only one which at once explains its act, as it occurs in the case of hernia of the stomach through the diaphragm, such as the one detailed by Dr. Graves and Dr. Stokes; and the experiment of M. Magendie, in which a bladder was substituted in the place of the stomach. The first establishes the fact, that the diaphragm, the second, that the stomach, has no necessary part in vomiting. It remained, therefore, to shew in what other manner the act of vomiting, and both of these facts, would admit of explanation. This is done in the manner already detailed: and the truth of the explanation is proved by two decisive experiments, related in the paper to which I have already referred.—*Journal of the Royal Institution.*

* In this paper I have referred to cases similar to that given by the authors of the Report.

### PATHOLOGY.

*Modes of ascertaining the Capacity of Diseased Lungs.* LAENNEC takes no notice of the mode of ascertaining the capacity of the lungs, proposed by Mr. ABERNETHY, and which may be named *Pulmometry*. This proposal I consider as highly ingenious, and as deserving much more attention than it has hitherto received. "Its object (I use the words of Dr. YOUNG,) is to measure the capacity of the remaining cavity of the chest, by means of a pneumatic apparatus. Inverting a large jar filled with water in a basin, the patient makes a deep inspiration, and blows into the jar through a bent tube: the height of the water remaining in the jar is then marked; and, by filling the bottom of the jar up to the mark, and measuring the fluid contained in it, we may easily ascertain the quantity of air which has been expired. This, in a healthy person, should be from six to eight quarts; and, if it is only one or two, we may be sure that the lungs, or some other parts concerned in respiration, are materially diseased."

A more simple test, on the same principle, has lately been proposed by Dr. LYONS, staff-surgeon, (Edinb. Journal, vol. xxviii. p. 453.) This consists in observing the comparative length of time occupied in making a complete expiration, after a complete inspiration. The patient, during expiration, is desired to count as far as he can, slowly and audibly. The number of seconds he is able to continue counting in the expiration, is noted by a watch. This time will, of course, *cæteris paribus*, bear a proportion to the integrity of the lungs. Dr. Lyons says, that the most healthy individual will not continue counting beyond thirty-five seconds. In confirmed phthisis, he says, the period never exceeds eight, and frequently is less than six or seven seconds; while in pleurisy and pneumonia, it may range from four to nine. This test is a much easier application than Mr. Abernethy's, but it is infinitely less accurate: it is liable to many obvious accidents, producing vitiation and fallacy.—*Note by* Dr. FORBES *in his Translation of* LAENNEC *on Diseases of the Chest.*

---

*Foreign Body in the Œsophagus;* by Dr. BOILEAU, of Nancy. A man had eaten and drank to excess at a village feast. Three or four hours after, he vomited a part of the food he had taken. Violent efforts to vomit continued for some time, but nothing more was thrown from the stomach. He complained of pain and obstruction in the course of the œsophagus. He could neither swallow his saliva, nor any drink that was offered to him. His respiration soon became laborious, and he could not speak distinctly: he carried his hand to his neck, and attempted to speak, but uttered only some inarticulate sounds. When M. B. arrived, the patient was in great distress, and threatened with instant suffocation. He shewed, by signs, that there was some obstruction in the throat. He was persuaded to make an attempt to swallow a little fluid, but it no sooner reached the pharynx than it was returned by the mouth and nostrils. It was presumed that there was in the œsophagus some foreign body, which prevented deglutition and speech, and which also impeded respiration. M. B. procured a piece of whalebone, about fourteen or fifteen inches long, four or five lines in breadth, and two in thickness. To the end he firmly fixed a piece of lint, covered with simple ointment. This instrument was passed into the œsophagus, and at the upper portion of the canal resistance was felt. By slight pressure downwards, the obstacle moved, and passed into the stomach. The patient cried out, the moment the instrument was withdrawn, "I am saved." He was immediately restored to a state of comfort.

This case is interesting, inasmuch as it shews that the œsophagus may be obstructed by a foreign body, which had not been introduced immediately by the mouth: but that a mass of food, after having remained some time in the stomach, may ascend, during attempts to vomit, and be arrested in the œsophagus, and produce the most alarming symptoms, if the case is misunderstood, and the most prompt and efficacious treatment is not applied.—*Arch. Gen.* Août. 1330.

---

*Case of Facial Neuralgia, cured by spontaneous Ptyalism.* By Dr. Caresi. A lady, of delicate constitution, fifty-two years of age, had experienced violent pain in the nerves of the face, for four months: the attack first commenced after a cold. Many vain attempts had been made to arrest the neuralgia, but several of the medicines employed appeared rather to aggravate the symptoms. The patient resolved to employ no more remedies, and to leave the cure ,to nature. Fifteen days elapsed without any improvement, when a copious secretion of saliva occurred spontaneously. The saliva was of a sweet odour, resembling honey, or the urine of diabetic patients; and increased in quantity, for three days, to such an extent, that the patient appeared to vomit it: it flowed from her mouth incessantly, when she spoke or slept with her mouth open; and when she slept with her mouth shut, she was threatened with suffocation. During the whole time, the bowels were confined, the urine and other secretions scanty.

---

### PRACTICAL MEDICINE.

*Extract of Gratiola (Hedge Hyssop) used in Delirium Tremens.*—An officer, thirty-two years of age, of a vigorous constitution, and always in the enjoyment of good health, had been in the habit of drinking very freely for six years. He had lately swallowed extraordinary quantities of brandy. One evening, after having eaten but very little for some weeks, he was attacked with severe headach, which was quickly followed by delirium, and the appearance of spectres before his eyes. These symptoms increased, and the following day leeches were applied to his head, and blisters to the calves of the legs, and a mild infusion of valerian was prescribed. No improvement occurred, and M. Mukebeck was consulted. He found the patient dressed and up, but reeling here and there, and stretching out his trembling hands towards the objects which, in his delirium, he imagined he beheld. He talked incessantly, and changed his subject abruptly. Sometimes he sought for arms to repel the enemy: then believed his chamber was on fire, &c. He occasionally knew the persons about him, but more frequently he confounded them with the imaginary spectres. His pulse was rapid, small, and hard; his look fixed and vacant; there was an increased and circumscribed redness of the face; tongue red, and towards the base covered with mucus. The patient had no appetite, but periodically a troublesome thirst, which he gratified by drinking small beer. Bowels constipated for forty-eight hours. No pain was felt from the blisters, although no dressing had been applied. No sleep since the commencement of the delirium.

Under these circumstances, it was impossible to mistake the nature of the disease: it was a sub-inflammatory delirium tremens. M. Mukebeck prescribed immediately three grains of extract of gratiola, with a scruple of tartrate of potash, to be taken in water every hour. At the expiration of seven hours, twenty-one grains of the extract having been taken, the patient became more calm. He was persuaded to go to bed, and in half an hour he fell fast asleep. He slept for seven hours, and awoke the next morning in full possession of his faculties. His bowels were freely evacuated: he remained perfectly conscious, and remembered nearly every circumstance that had happened during the delirium. From this time no medicine was given: he drank some coffee, and arose from bed with little to complain of except debility.

When this case of delirium tremens is compared with others in which opium has proved efficacious, it must be admitted that the nature of this disease is very variable, and that the treatment must necessarily be varied accordingly, if we wish to treat it successfully. We must abstain from administering opium when the character of the delirium manifests an exalted state of the corporeal powers, and when the patient shews less timidity than excessive and violent paroxysms of passion; when the trembling of the hands appears to be rather a sthenic than asthenic spasm, and when there are general signs of an inflammatory diathesis. In all these cases opium will be injurious, while the extract of gratiola in general acts as a specific.—Hufeland's *Journal*, July 1830.

Of the remedy so warmly recommended by M. Mukebeck, we know nothing from our own experience. We are not aware, indeed, that it is now ever used in this country. It may be worth while to mention, that M. Kostrzewski states that, in the Vienna hospitals, three maniacal patients were perfectly recovered by it. The above account is principally interesting from the distinction which is made between those cases of delirium tremens in which opium is likely to be useful, and those in which it is more than probable it will aggravate rather than relieve the symptoms. We have reason to know that the very surprising effects of opium, in appropriate cases of this malady, have sometimes led to its use where the general habit of the patient, and the symptoms of his attack, strongly contra-indicated its employment. Let the young practitioner, therefore, be on his guard, and not imagine that opium is the remedy required in *all* cases of delirium tremens.—EDITOR.

---

*Belladonna in Hydrophobia.* A case of hydrophobia is related in the last volume of the Transactions of the Medical and Physical Society of Calcutta, in which twenty-three grains of belladonna were given in twenty-six hours, without any apparent benefit. The patient was attended by Mr. BAKER and Mr. LEESE. The former gentleman, in his detail of the case, observes, that he would in future commence with ten grains of the extract, and repeat it, in larger quantities, every two or three hours, if it did not produce any decided effect.

It appears by Mr. BRODIE's experiments, (ORFILA on Poisons, ii. p. 197,) that twenty grains of extract of belladonna were given to a cat, and that, though it retained about two thirds of this quantity, in five hours it was perfectly recovered; and that, though the pupils were dilated, and the animal appeared intoxicated, it still preserved its sensibility. By another experiment, four drachms of the extract were given to a dog at half-past one, and he did not die till nine o'clock the following evening.

---

### SURGERY.

*Excision of Cancer of the Rectum.* M. LISFRANC recently presented to the Académie Royale de Médecine, a memoir upon the Excision of the lower Portion of the Rectum, when carcinomatous. Having proved, 1st, that the peritoneum does not cover the six inferior inches of the rectum in females, and the four inferior inches in males; 2d, that no great inconvenience arises from dividing the numerous vessels upon the lower portion of the rectum; 3d, that, by means of an oval incision in the skin around the anus, the rectum is easily drawn out; 4th, that there exists a second sphincter above the first; M. Lisfranc conceived the possibility of a salutary operation in such cases: but little is to be apprehended from consecutive inflammation, which is known to be very rare after the operation for fistulæ ani, &c. He commenced by excising superficial cancer, which only implicated the mucous membrane of the rectum; he then operated upon cases in which the sphincters were partly diseased; lastly, he removed about three inches and a half of the rectum.

He recommends the operation in all instances when the index finger can be passed beyond the limits of the disease, and when the cellular tissue external to the rectum is healthy. As guides in the performance of the operation, the following facts should be borne in mind: 1st, that the anterior posterior diameter of the perineum is generally one inch; 2d, that the distance from the anus to the coccyx is eighteen lines, and the interval between the anus and the base of that bone two inches; 3d, that, both laterally and posteriorly, sufficient portions of the rectum may be removed, without wounding the vagina in the female, or the urethra in the male; 4th, that it is always possible to arrest hemorrhage from the operation, by compression or ligatures. The patient should be placed in the same position as for the lateral operation for the stone; two semi-lunar incisions are to be made around the anus; the inferior extremity of the rectum is insulated; by means of the index finger introduced into the intestine, it is drawn out, and divided with a pair of scissors. At first, light dressings should be laid upon the wound, but afterwards a roll of lint is to be introduced into the rectum. After the parts are healed, the fæces are sometimes passed in the natural manner, an internal ring being formed, which performs the duties of a sphincter. Occasionally liquid fæces cannot be retained, and the patient may be obliged to plug the intestine with lint.

M. Lisfranc has operated six times with success: in three instances the patients died on the third, fourth, and the twenty-fifth day after the operation.

---

### MIDWIFERY.

*Abdominal Pressure in Obstetrics.* Mr. M'Morris, a medical officer in the East-India Company's service, writes to us as follows:

"In one of the numbers of the Medico-Chirurgical Review for 1828, I find a bandage proposed by Mr. Gaitskell for obstetric purposes. I have no doubt it will facilitate the parturient powers of nature, by the support and pressure it affords. It is a common auxiliary among the native women on this side of the Indian peninsula, when the progress of labour is slow. Some of the female attendants, in such cases, place their hands on the abdomen of the parturient patient, and press it pretty violently downwards towards the pubes, having first rubbed it with castor oil. This operation is continued till the child is expelled, which generally happens soon after the mechanical pressure is commenced. If the secundines are not expelled with the fœtus, the woman is left to rest, with a bandage applied pretty tightly round the abdomen. I have been astonished, in some cases that I have witnessed, to observe the increase of the expulsive pains, soon after this process is put in force." *Delhi*, 1830.

"N.B. The above procedure corresponds with that recommended by Dr. Power, a few years ago, and which was, at the time, too slightingly entertained by our obstetric brethren."—*Ed. Med.-Chir. Rev.*

